# Complete functional recovery in a child after endovascular treatment of basilar artery occlusion caused by spontaneous dissection: a case report

**DOI:** 10.1007/s00381-021-05428-w

**Published:** 2021-12-10

**Authors:** Ljubisa Borota, Sylwia Libard, Markus Fahlström, Francesco Latini, Erik Lundström

**Affiliations:** 1 Department of Surgical Sciences, Radiology, Uppsala University, Uppsala, Sweden; 2 Department of Pathology, Uppsala University, Uppsala, Sweden; 3 Department of Neuroscience, Uppsala University, Neurosurgery, Sweden; 4 Department of Neuroscience, Uppsala University, Neurology, Sweden

**Keywords:** Pons, Infarction, Diffusion tensor imaging

## Abstract

Stroke caused by dissection of arteries of the vertebrobasilar system in children is still poorly investigated in terms of etiology, means of treatment, course of disease, and prognosis. The aim of this report was to describe the unusual course of a spontaneous dissection of the basilar artery (BA) in a child treated with endovascular techniques and to point out that the plasticity of the brain stem can fully compensate for structural damage caused by stroke. We report the case of a 15-year-old boy who suffered a wake-up stroke with BA occlusion caused by spontaneous dissection. A blood clot was aspirated from the false lumen and the true lumen re-opened, but the patient deteriorated a few hours later, and repeated angiography revealed that the intimal flap was detached, occluding the BA again. The lumen of BA was then reconstructed by a stent. Despite a large pons infarction, the patient was completely recovered 11 months after the onset. The case was analyzed with angiograms and magnetic resonance imaging, macroscopic and microscopic pathological analysis, computed tomographic angiography, magnetic resonance-based angiography, and diffusion tensor imaging. This case illustrates that applied endovascular techniques and intensive care measures can alter the course of potentially fatal brain stem infarction. Our multimodal analysis gives new insight into the anatomical basis for the plasticity mechanism of the brain stem.

**Supplementary Information:**

The online version contains supplementary material available at 10.1007/s00381-021-05428-w.

## Introduction

A spontaneous dissection of arteries of the vertebrobasilar system in children is a rare clinical condition, and a dissection which involves only the basilar artery (BA) with intact vertebral arteries is even more uncommon [1–3]. In the posterior circulation, conservative treatment has been the standard approach in patients with spontaneous dissections [4]. However, endovascular (EV) interventions are being increasingly used in the treatment of dissections of vertebral and basilar arteries even in pediatric patients [5].

## Case report

**Fig. 1:**
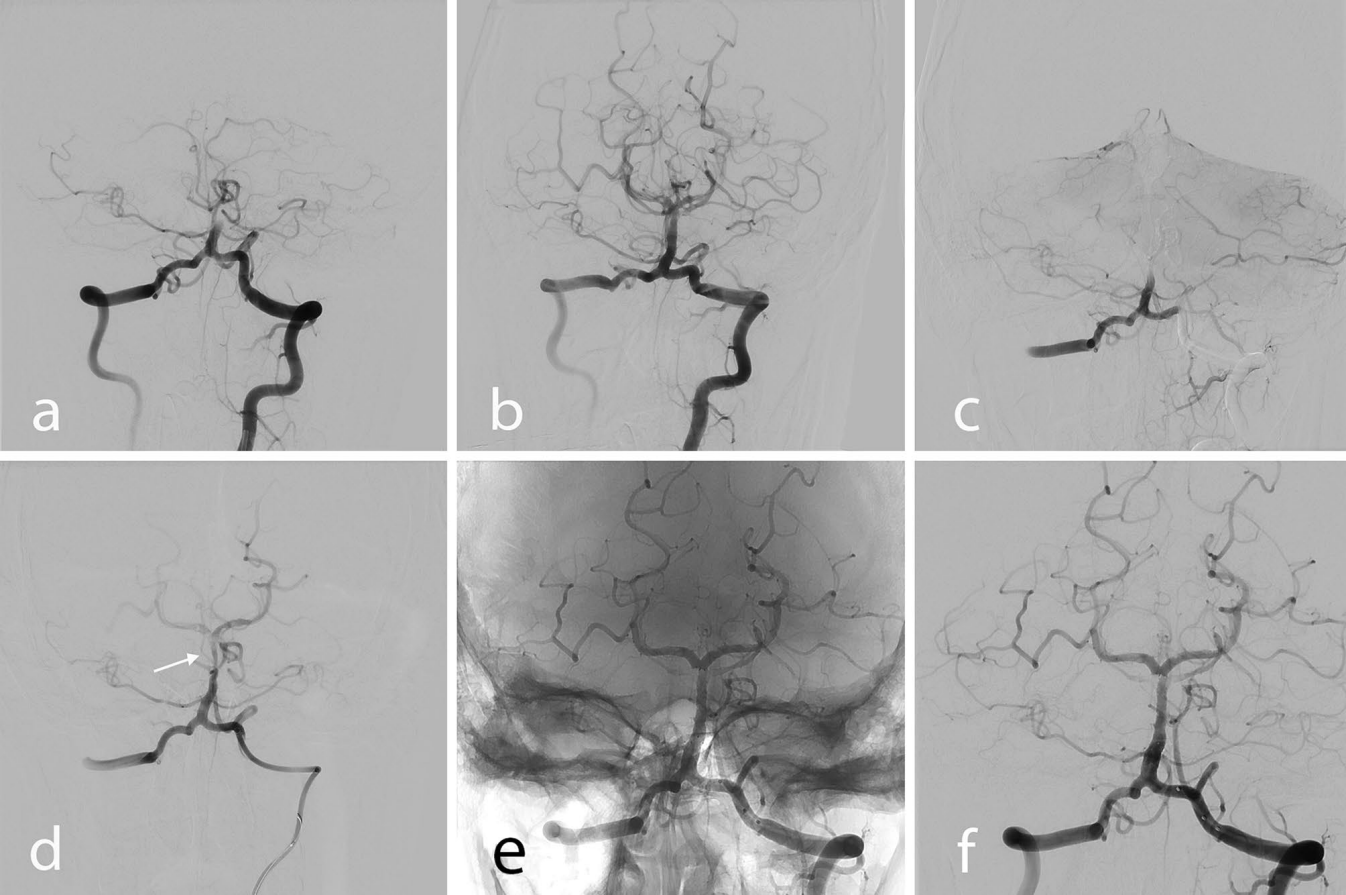
**a** and **b** First intervention, angiography of basilar artery before (**a**) and after recanalization (**b**). **c** and **d** Second intervention. Angiography shows detached intimal flap which closes the basilar artery completely (**c**) or partially (**d**, arrow). **e** Non-subtracted spot image shows stent deployed in the basilar artery. **f** Angiography, last run, shows reconstructed basilar artery and normal flow in both posterior cerebral arteries. Slightly irregular, the narrowed lumen in both superior cerebellar arteries indicates that the dissection has progressed to both these arteries

A previously healthy 15-year-old boy developed headache and vomited late in the evening after an ordinary day without any unusual physical activity. The body temperature was normal. He fell asleep quickly, and his parents did not notice anything unusual during his sleep. The next morning, it was not possible to wake him. Upon arrival at the hospital, he presented with right-sided weakness, dysarthria with a National Institutes of Health Stroke Score of 8 [6]. A computerized tomographic angiography (CTA) showed occlusion of the BA at the level of anterior inferior cerebellar arteries (AICAs). The boy was quickly intubated. The CTA finding was confirmed with conventional angiography (Fig. 1a). A coaxial system consisting of 8F Infinity long sheath (Stryker, Fremont, CA, USA) and 6F SOFIA distal access catheter (DAC) (Microvention, Aliso Viejo, CA, USA) was inserted up to the BA. The DAC was then navigated over a Transend Extra Support Guidewire (Stryker, Fremont, CA, USA) to the obstruction. The DAC was connected to a pump. A dark-red, fine granular material looking like sludge was aspirated immediately after the start of pumping and sent for histological examination. The control angiography showed that the BA was open, but with slightly irregular contours (Fig. 1b). The clinical condition of the patient was stable but did not improve after restoration of the flow in the BA. Five hours after the intervention, the patient suddenly deteriorated. A new CTA showed an occlusion of the BA at the same level—the origin of AICAs and the procedure described above were repeated. This time, the same combination of introducer, long sheath, and catheters was used. The occlusion site was passed easily with the SOFIA DAC. The continuous injection of contrast during the slow withdrawal of this catheter revealed pronounced irregularity of the wall and intimal flap indicating a dissection (Fig. 1c and d). The BA was then stented with a 3 × 20 Acclino HRF stent delivered through a NeuroSpeed Balloon microcatheter (Acandis, Pforzheim, Germany) (Fig. 1e). Before deployment of the stent, the patient was given 400 mg acetylsalicylic acid I.V. and a single weight-adjusted dose of abciximab. The last run showed a reconstructed lumen of the BA and flow in all its branches but also narrowed origins of superior cerebellar arteries indicating involvement of even these arteries by dissection (Fig. 1f). The patient was on dual antiplatelet therapy from day 2, consisting of 75 mg aspirin daily for 12 months and 180 mg of Ticagrelor (Astra Zeneca AB, Södertälje, Sweden) divided into 2 doses daily for 6 months.

Initial neurologic wake-up tests revealed a Glasgow coma score of 11. The patient was treated at neurointensive care unit, with close monitoring of neurological status and systemic vital functions using local standard operating protocols to prevent secondary brain damage. The patient was sedated with propofol and fentanyl. Initially, the patient required some support with norepinephrine, but there were no major intensive care problems. However, the patient developed pneumonia, which was treated with cefotaxime and was probably a contributing cause for re-intubation. During the first week, the prognosis looked gloomy, due not only to major swallowing problems but also to tetraplegia. The patient gradually improved and could be extubated after 7 days. Because of a paralysis of the vocal cords and to better manage airway secretions, the patient received a tracheostomy. After extubating, the patient was transferred to the Intermediate neuro care unit, where he spent 2 weeks. He was then transferred to The Department of Pediatric Neurology, where he spent 4 weeks more. The tracheostomy was closed 2 weeks before discharge. The clinical condition of the patient improved significantly during this period, and he could be discharged 7 weeks after onset of the disease. At discharge, the patient was able to perform all usual duties and activities despite some minor symptoms. In other words, his neurological status at discharge corresponded to level 1 on the modified Rankin scale.

During the following months, the patient underwent standard rehabilitation.

At a face-to-face follow-up after 11 months, the patient was fully recovered, with a score of 0 on both the National Institutes of Health Stroke Scale [6] and the modified ranking scale [7].

### Pathological findings

**Fig. 2:**
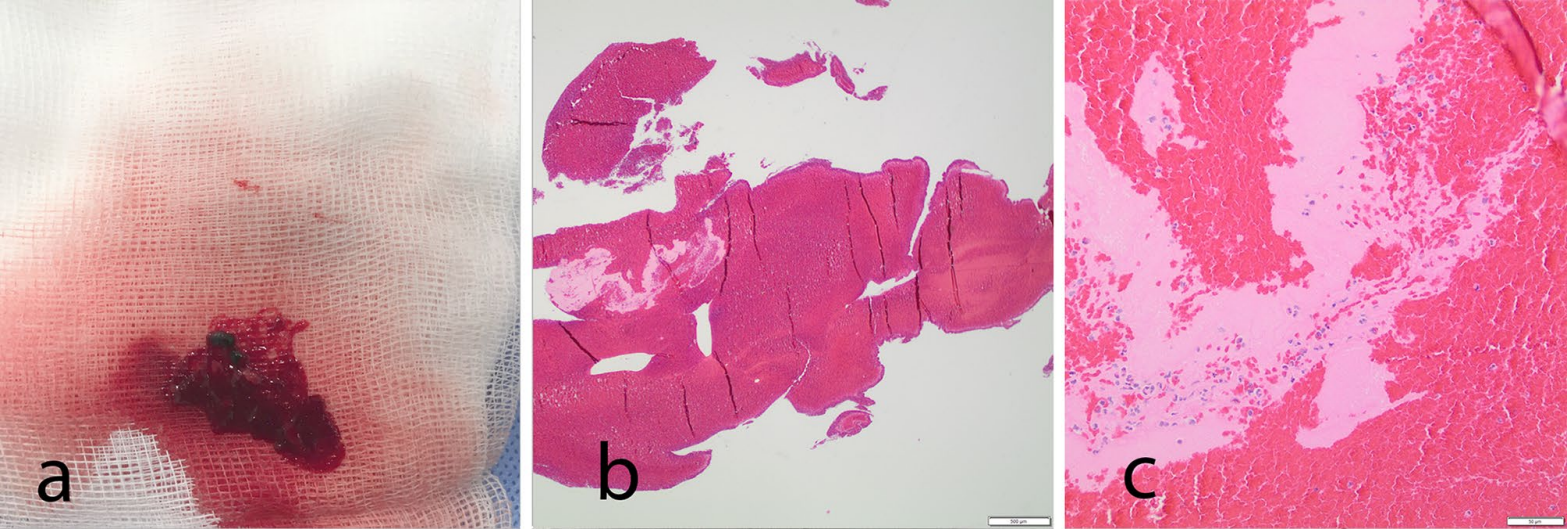
**a** Macroscopical photo of the specimen aspirated from the basilar artery during the primary intervention. **b** Hematoxylin–eosin (HE)-stained section of the specimen showing fresh hematoma with early clot formation, composed mainly of erythrocytes and a focal fibrin deposition. Bar 500 µm. **c** HE-stained section visualizing the loose fibrin matrix within the hematoma, which contained a few scattered neutrophilic granulocytes. Bar 50 µm

All material received was included for further analysis. Microscopic examination of the aspirated material revealed fragments of a fresh hematoma with an early clot formation, composed mainly of erythrocytes with focal fibrin depositions and some scattered neutrophilic granulocytes. An organized thrombus was not seen in the examined sample (Fig. 2).

### Postoperative course

**Fig. 3:**
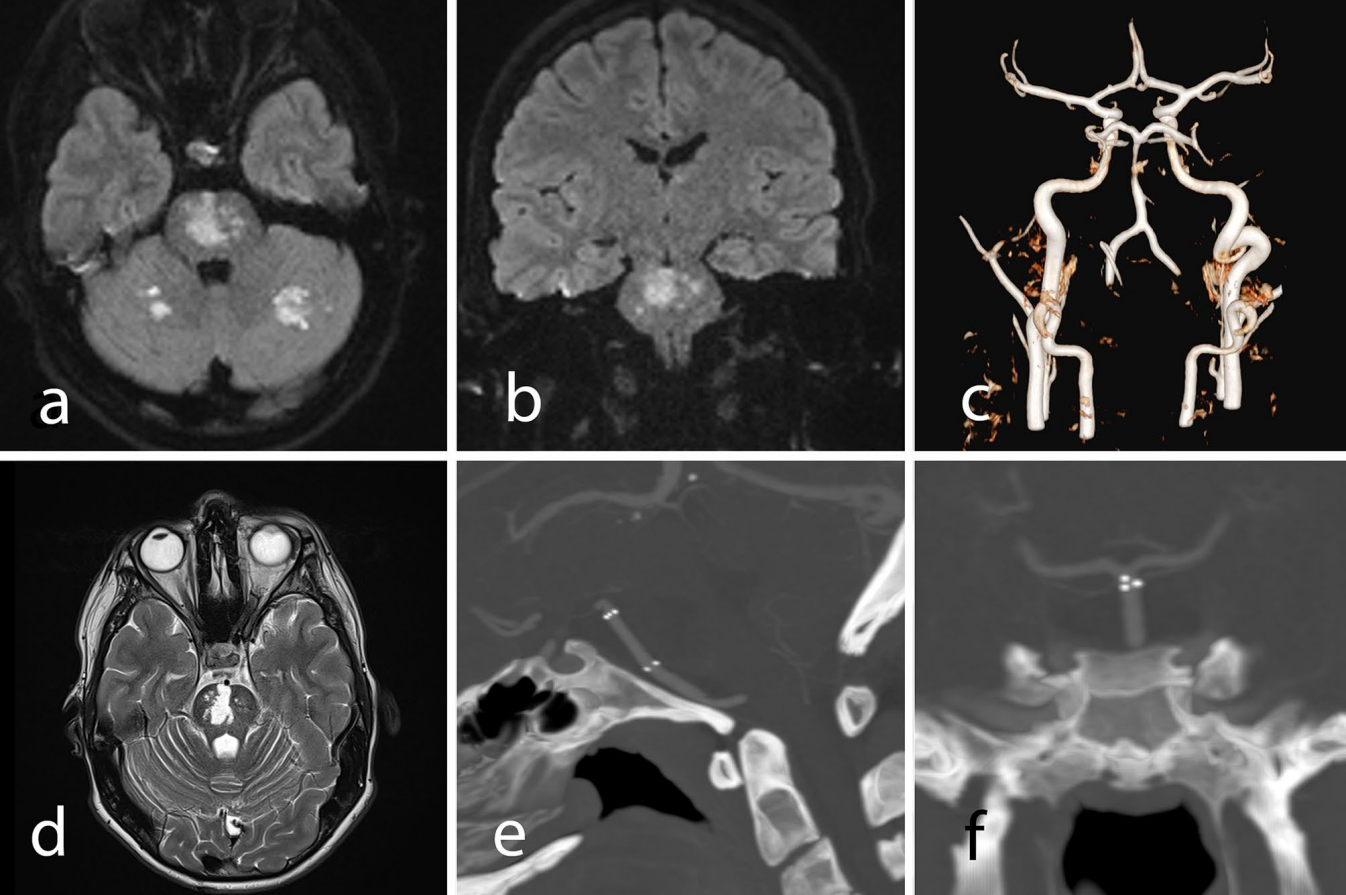
**a** and **b** Day 1 follow-up MRI: diffusion-weighted images show acute infarction involving the upper third of pons. Signs of fresh ischemia in both cerebellar hemispheres. **c** Day 1 follow-up MR angiography, 3D reconstruction, shows flow in basilar artery and its branches. The lumen is only seemingly narrowed—a consequence of the artifacts caused by a metal stent inserted into the basilar artery. **d** 1-month follow-up MRI shows post-ischemic, fluid-filled cavity occupying more than one-third of cross-section of the upper pons. **e** and **f** 10-month follow-up, CT angiography shows stent in basilar artery, reconstructed lumen of the basilar artery, and normal flow in all its branches

MRI performed the day after the intervention showed multiple bilateral cerebellar infarctions and a centrally located, symmetric infarction occupying approximately 50% of the cross-section of the pons and extending from the level of the superior cerebellar arteries to the level of AICAs (Fig. 3a, b). MR-based angiography (MRA) showed flow in the BA and all its branches (Fig. 3c). MRI performed 1 month after the procedure showed multiple fluid-filled cavities in the pons corresponding to post-infarction malacia (Fig. 3d). CTA 10 months after the procedure showed a reconstructed BA and flow in all its branches (Fig. 3e, f).

**Fig. 4:**
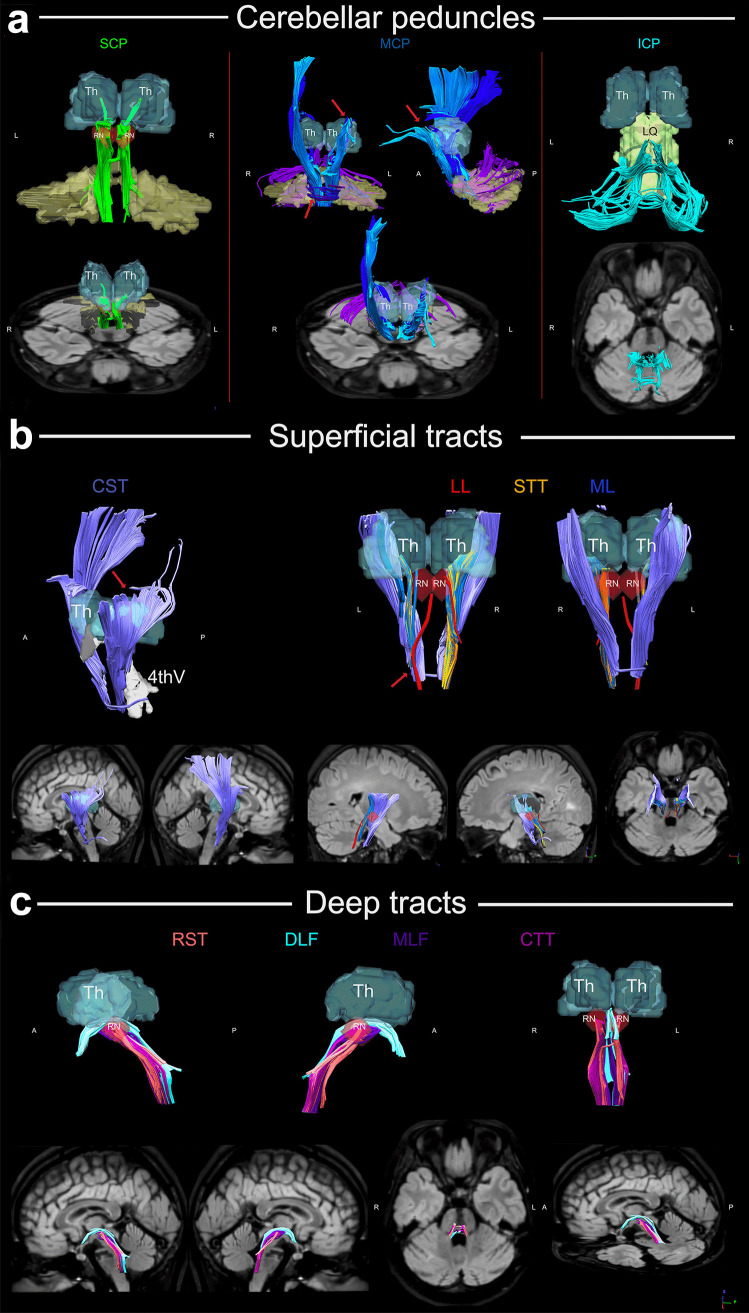
**a** The image illustrates the 3D reconstruction of the three cerebellar peduncles (upper part) and their relationship with the ischemic lesion on T2-FLAIR images (lower part). The superior cerebellar peduncle (SCP, in green) includes the dento-rubro-thalamic tract with ipsilateral and crossing fibers originating from the red nucleus bilaterally. From the red nucleus (RN), each tract ascends toward the thalamus (Th). The middle cerebellar peduncle (MCP) is formed by the fronto-pontine tract (sky color) and parieto-pontine tract (blueberry) and transverse fibers originating from pontine nuclei crossing the whole pons (grape color). The red arrows indicate the asymmetric reconstructions and the suspected damage due to the ischemic lesion within both close (transverse fibers) and remote (fronto-parieto-pontine tracts) areas. The T2-FLAIR images show the spatial relationship between the ischemic lesion and the partially reconstructed tracts. The inferior cerebellar peduncle (ICP) fibers ascend dorsolaterally to the roof of the fourth ventricle and partially distribute to the upper vermian cortex. The largest component of the ICP encircles the fastigial nuclei from medial and superior to lateral and inferior and reaches the inferior vermis and the posterior cerebellar lobe and flocculus. **b** The image illustrates the 3D reconstruction of the superficial white matter tracts (upper part) and their relationship with the ischemic lesion on 3D-FLAIR images (lower part). The cortico-spinal tract (orchid color) displayed possible remote damage to the left side, superior to the lesion level (red arrow). Relationship with the fourth ventricle (4th V) and the thalamus bilaterally is shown. The lateral lemniscus (LL, red), medial lemniscus (ML, ocean), spino-thalamic tract (STT, orange) are displayed from both an anterior and posterior prospective. The left STT was incomplete and possibly damaged (red arrow). **c** The image illustrates the 3D reconstruction of the deep white matter tracts (upper part) and their relationship with the ischemic lesion on T2-FLAIR images (lower part). The rubro-spinal tract (RST, salmon), the dorsal longitudinal fasciculus (DLF, ice), the medial longitudinal fasciculus (MLF, eggplant), and the central tegmentum tract (CTT, plum) were reconstructed without apparent sign of damage. The relationship with the thalamic red nucleus is displayed on 3D reconstruction. LQ, lamina quadrigemina

DTI data obtained 10 months after the procedure were reconstructed in the MNI space using q-space diffeomorphic reconstruction [8]. For a detailed description of image acquisition and processing, see supplementary material 1. Three groups of white matter fibers crossing the brain stem were reconstructed as described by Meola et al. [9]: three cerebellar peduncles (superior, middle, and inferior cerebellar peduncles), superficial white matter tracts (cortico-spinal tract, lateral lemniscus, medial lemniscus, and spino-thalamic tract), and deep white matter tracts (rubro-spinal tract, medial longitudinal fasciculus, dorsal longitudinal fasciculus, and central tegmental tract) (Fig. 4). In the first group, the middle cerebellar peduncle displayed signs of possible damage, with incomplete/asymmetric reconstructions of the fronto-pontine and parieto-pontine tract on the left side and the transverse fibers through the middle of the pons (Fig. 4a). Among the superficial white matter tracts, the cortico-spinal tract on the left side (dorsally, beyond the internal capsule), and the spino-thalamic tract on the left side displayed signs of incomplete/asymmetric reconstruction (Fig. 4b). No other damages/asymmetries were found among the deep tracts (Fig. 4c).

## Discussion

### Neurointervention

The first report of BA occlusion due to spontaneous dissection of BA in a child, with all relevant radiological and clinical data and follow-up from onset of the disease to the outcome, was published in 1999 [10]. Songsaeng et al. reported on eight children, aged between 1 and 12 years, with spontaneous dissections of intracranial arteries, three of them with spontaneous dissection of the BA. All patients were treated conservatively. Ischemic lesions in the pons were revealed in only one of these three patients. Of two patients without identified ischemic lesions, one patient had recovered completely at the end of clinical follow-up [4]. The same group reported on 29 children with vertebrobasilar dissections in which, besides the conservative treatment, open surgical and EV techniques were applied with varying results [11].

EV clot retrieval in the treatment of acute stroke in young children has recently been more widely reported in the literature due to the promising results obtained with this technique in the treatment of stroke in adults. According to the review by Sun et al., the endovascular treatment of stroke in younger children (< 5 years) was applied previously very sporadically [12]. Hutchinsson et al. treated 90 children (aged 2–18) with stroke in the period between 2005 and 2017 and applied endovascular treatment in only two cases [13]. Mastrangelo et al. specified inclusion criteria for endovascular stroke treatment in children [14]. The technique of endovascular treatment of stroke in children and adults is principally the same, but differences in vascular anatomy in these two groups of patients (vessel size, anatomy of collateral vessels) influence the choice of thrombectomy devices [15]. Sporns et al. analyzed results of two different types of stroke treatment in a pediatric population: a direct aspiration first pass technique (ADAPT) and a non-ADAPT. The results revealed no difference with regard to the success of recanalization as assessed by the Pediatric National Institutes of Health Stroke Scale on day 7 after onset of symptoms/treatment and the modified ranking scale during and at the end of the follow-up period [16]. In this retrospective, observational, multicenter cohort study (27 centers in the USA and Europe), 73 patients with a median age of 11.3 years were included. Posterior circulation stroke with occlusion of the basilar artery as a cause of the stroke was recorded in only eight cases. None of these cases was stenting of the basilar artery applied. The general consensus is that EV treatment of stroke in children has been established as a method of choice in selected cases [16].

Our unique case is an example of a wake-up stroke where two different therapeutic EV techniques were applied several hours apart. Retrospective analysis helped us to reconstruct the evolution of pathological changes in the wall of the BA between the two interventions. The coagulated blood was aspirated from the false lumen of the dissection, which was widely opened toward the vertebrobasilar junction. After aspiration, the false lumen collapsed and the true one reopened. Even though the contours of the BA were irregular, we refrained from deploying a stent because the dissection was not clearly visible, and the flow in the BA and its branches had been re-established. The dissection progressed after aspiration and involved large segment of the BA, which resulted in dissection of the artery and formation of a free intimal flap, which unfolded like a parachute and occluded the BA again. At this juncture, the only therapeutic option was deployment of a stent.

### Histopathological findings

The aspirated coagulated blood corresponded neither in form or consistency to a thrombus, which was further supported by microscopical examination. Here, we observed mainly bleeding with sparse fibrin deposition and a few neutrophils, a finding in line with what is seen in acute hemorrhages or hematomas with early clot formation [17, 18]. Even though thrombus composition in cerebral arteries is heterogeneous, there were no signs of organization in the specimen assessed in this case, and the fibrin deposition was focal and sparse and thus did not fulfill the criterion for a thrombus [19].

#### MRI

Neurological recovery after stroke depends mainly on the location of the lesion. However, patterns of neural plasticity following brainstem ischemia are almost unknown [20, 21]. DTI has been used to characterize Wallerian degeneration after basal ganglia and brainstem strokes and to attempt to understand the structural basis for potential recovery [22, 23]. Even though the area of damage in our patient, involving the pons, is not commonly considered a silent or a surgical safe entry zone [9, 24, 25], the brain seems to be able to compensate for structural damage to supra-infra tentorial white matter connectivity. The observed damage/incomplete neuronal loss to fronto-pontine, parieto-pontine, and transverse fibers of the middle cerebellar peduncle and spino-thalamic tract may indicate a direct consequence of the brainstem ischemia, with complex cellular and axonal changes near to the lesion and in remote brain areas [20, 22, 23]. Even the damage to cortico-spinal tract superior to the lesion level, observed in our patient, has previously been described as remote damage in both human and animal models [20, 23]. However, as previously described, neurological functions gradually improve over time in patients with brainstem ischemia [26, 27]. Young age is an important factor influencing the plastic potential and the response to rehabilitation [27]. On the other hand, the position of the brainstem ischemia also seems to be important [20, 23, 26]. The dorsal brainstem plays a key role in sustaining proliferation of neural precursor cells leading migration of such newly generated cells toward the ischemic lesion [20, 28]. An intact dorsal brainstem combined with strengthening of ipsilesional cortico-rubral connections is an anatomical route by which neurons of the unaffected motor cortex gain control over the injury-denervated spinal hemicord and thus improve functional recovery [20, 28]. In our patient, the dorsal brainstem, the dento-rubro-thalamic tract, and the rubro-spinal tract were not affected by the ischemic lesion and were in fact reconstructed with both ipsilateral and crossing fibers. Our results and models of brainstem plasticity suggest that the younger age, the intact dorsal brainstem, and the known compensatory role of extrapyramidal pathways after pyramid fiber damage through bilateral cortico-rubral tracts may explain the complete recovery after pontine ischemia in our patient [20, 22, 28].

## Conclusion

As exemplified in our case, the course of BA occlusion is unpredictable. Despite a huge pons infarction and the two different interventions a few hours apart, the neurological outcome was surprisingly favorable in this patient. Our analysis of DTI, performed after the patient, had fully recovered and helped us to identify some of the structures in the dorsal pons that may play a key role in compensating for the destruction of the tracts in the ventral pons. Studies on the correlation between brain stem infarctions, applied endovascular treatments, damage to the tracts, compensation for this damage by the intact structures, and the evolution of the clinical picture have not been carried out previously. Further studies, including multimodal analysis in large numbers of patients, may reveal in detail the neuroplasticity potential of the brainstem. These results could contribute to the optimization of brainstem infarction treatment protocols in both children and adults.

## Supplementary Information

Below is the link to the electronic supplementary material.

Supplementary file1 (DOCX 20 KB)
