# Supplementary material for: Complete functional recovery in a child after endovascular treatment of basilar artery occlusion caused by spontaneous dissection: a case report
Source: Childs Nerv Syst. 2021 Dec 10;38(8):1605–12. doi: 10.1007/s00381-021-05428-w (PMC9325841; doi:10.1007/s00381-021-05428-w)
Supplement: Supplementary file 1 — Supplementary file1 (DOCX 20 KB) [file 381_2021_5428_MOESM1_ESM.docx]

Electronic Supplementary Material 1

The diffusion images were acquired on a Philips IngeniaElitionX scanner using a diffusion sequence with TE=93.389 ms, and TR=3925.73 ms. A DTI diffusion scheme was used, and a total of 32 diffusion sampling directions were acquired. The b-value was 800 s/mm2. The in-plane resolution was 1.75 mm. The slice thickness was 2 mm. The b-table was checked by an automatic quality control routine to ensure its accuracy (Schilling et al., 2019). The diffusion data were reconstructed in the MNI space using q-space diffeomorphic reconstruction (Yeh et al., 2011) to obtain the spin distribution function (Yeh et al., 2010). A diffusion sampling length ratio of 1.25 was used The output resolution of is 2 mm isotropic. The restricted diffusion was quantified using restricted diffusion imaging (Yeh et al., 2017). The fiber tractography was performed after positioning the region of interest in agreement with the known course of each tract, as described by Meola et al. (Meola et al., 2016). 3D-FLAIR T2-weighted sequence slices of the patient were normalized to MNI space and used to assist the placement of regions of interest and interpretations of tracts location and trajectory.

References

Meola, A., Yeh, F.-C., Fellows-Mayle, W., Weed, J., Fernandez-Miranda, J.C., 2016. Human Connectome-Based Tractographic Atlas of the Brainstem Connections and Surgical Approaches. Neurosurgery 79, 437–455. https://doi.org/10.1227/NEU.0000000000001224

Schilling, K.G., Blaber, J., Huo, Y., Newton, A., Hansen, C., Nath, V., Shafer, A.T., Williams, O., Resnick, S.M., Rogers, B., Anderson, A.W., Landman, B.A., 2019. Synthesized b0 for diffusion distortion correction (Synb0-DisCo). Magn Reson Imaging 64, 62–70. https://doi.org/10.1016/j.mri.2019.05.008

Yeh, F.-C., Liu, L., Hitchens, T.K., Wu, Y.L., 2017. Mapping immune cell infiltration using restricted diffusion MRI. Magn Reson Med 77, 603–612. https://doi.org/10.1002/mrm.26143

Yeh, F.-C., Wedeen, V.J., Tseng, W.-Y.I., 2011. Estimation of fiber orientation and spin density distribution by diffusion deconvolution. Neuroimage 55, 1054–1062. https://doi.org/10.1016/j.neuroimage.2010.11.087

Yeh, F.-C., Wedeen, V.J., Tseng, W.-Y.I., 2010. Generalized q-sampling imaging. IEEE Trans Med Imaging 29, 1626–1635. https://doi.org/10.1109/TMI.2010.2045126
